# 9-O Acetylated Gangliosides in Health and Disease

**DOI:** 10.3390/biom13050827

**Published:** 2023-05-12

**Authors:** Luis Vicente Herrera-Marcos, Dil Sahali, Mario Ollero

**Affiliations:** 1 Univ Paris Est Creteil, INSERM, IMRB, F-94010 Creteil, France; luis.herrera-marcos@inserm.fr (L.V.H.-M.); dil.sahali@inserm.fr (D.S.); 2 AP-HP, Hôpitaux Universitaires Henri Mondor, Service de Néphrologie, F-94010 Creteil, France

**Keywords:** glycosphingolipid, sphingolipid, acetylation, cancer, sialic acid

## Abstract

Glycosphingolipids comprise a lipid class characterized by the presence of sugar moieties attached to a ceramide backbone. The role of glycosphingolipids in pathophysiology has gained relevance in recent years in parallel with the development of analytical technologies. Within this vast family of molecules, gangliosides modified by acetylation represent a minority. Described for the first time in the 1980s, their relation to pathologies has resulted in increased interest in their function in normal and diseased cells. This review presents the state of the art on 9-O acetylated gangliosides and their link to cellular disorders.

## 1. Discovery and Chemistry

**Figure 1:**
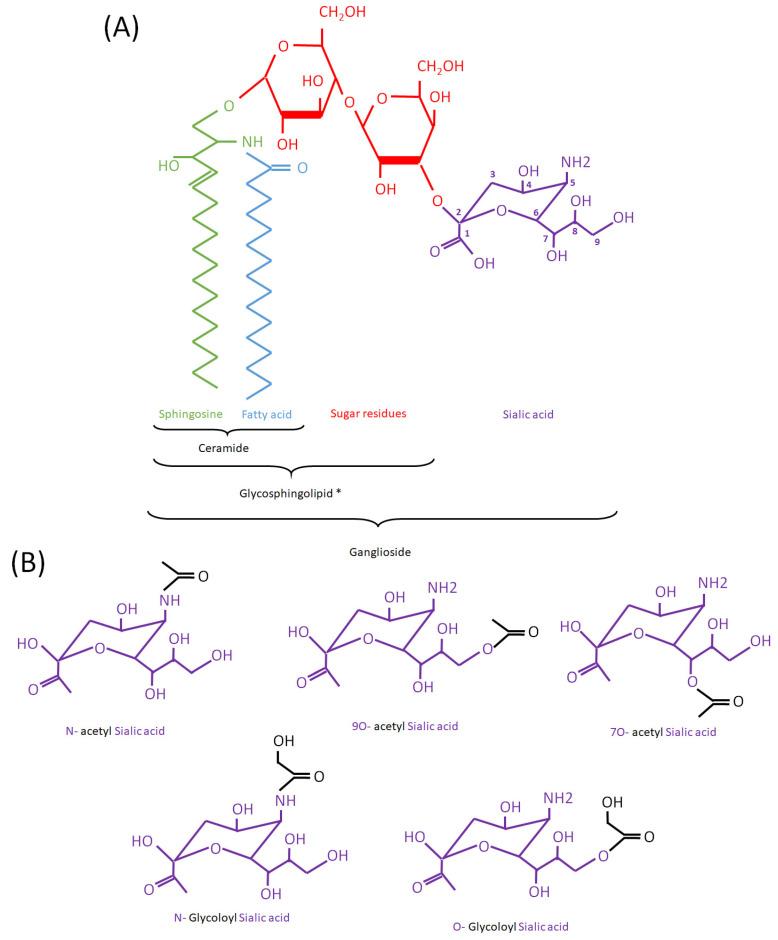
(Acetyl/Glycoloyl)-Ganglioside structure. (**A**): Schematic representation of GM3 as an example of ganglioside. * To note: gangliosides are a type of glycosphingolipid but neither gangliosides nor glycosphingolipids are considered as types of ceramides. Ceramide is a structural component of all glycosphingolipids (including gangliosides). Sialic acid carbons are numbered as 1 to 9, starting from the left side of the molecule. (**B**): Different types of sialic acid modifications in mammalian gangliosides mentioned in the text. N-acetylated (acetyl groups, in black, bound to the N atom) and O-acetylated forms (bound to an O atom) are represented on the upper part. A N-glycoloylated (a glycoloyl group, in black, bound to the N atom) and O-glycoloyl (bound to the O atom) are represented on the lower part.

Glycosphingolipids constitute a subcategory of sphingolipids in which a ceramide backbone is linked to one or more sugar residues. Among glycosphingolipids, gangliosides contain at least one residue of sialic acid, anciently known as neuraminic acid (Figure 1). Gangliosides are subdivided according to the number of sialic acid residues, e.g., monosialylated (GM), disialylated (GD), and trisialylated (GT), and further classified according to the number of neutral sugar residues subtracted from a maximum of five (e.g., GD1 contains four neutral residues, where “1” indicates 5-4 = 1) (Table 1). The sialic acid moiety contained in the ganglioside molecule can present structural modifications, such as acetylation. This modification can be present in other biomolecules containing sialic acid residues, such as glycoproteins.

### 1.1. Types of Acetylation and First Findings in Cells

**Figure 2:**
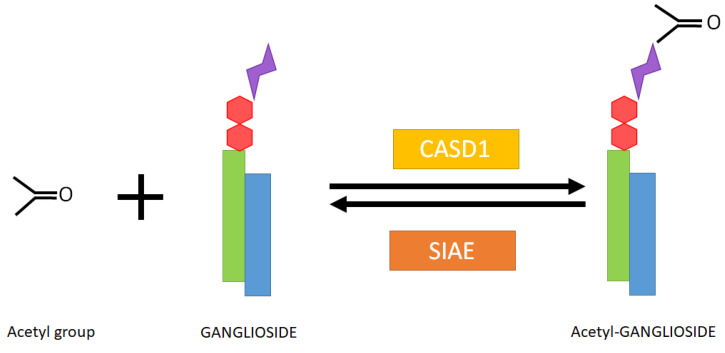
Enzymatic conversion of a ganglioside to its acetylated form and responsible enzymes in humans. CASD1: CAS1 domain containing (Uniprot ref. Q96PB1). SIAE: Sialate O-acetylesterase (Uniprot ref. Q9HAT2). Green rectangle: sphingoid base. Blue rectangle: Fatty acyl chain. Red hexagons: neutral sugar residues. Purple double triangle: sialic acid residue.

Modifications of sialic acid were first discovered in the secreted products of submandibular glands from cattle [1]. Those include O-glycoloyl, N-glycoloyl, O-acetyl and N-acetyl forms, where glycoloyl and acetyl groups are formed by the hydroxylation and acetylation of sialic acid, respectively (Figure 1). The acetyl and glycoloyl transferase activities necessary to ensure these modifications were found in cytosolic and microsomal extracts from these tissues [2,3]. The O-acetyl transferase reaction conveying the acetyl group to the sialic acid moiety (sialate O-acetyl transferase (SiAOAT) activity) has been recently attributed to the enzyme CASD1 (CAS1 domain containing) by means of genome-editing approaches [4]. This acetylation can be reversed by the 9-O-acetylesterase or sialidase activity (SIAE), found in several microorganisms and mammal brain tissue and resulting in the release of acetyl residues (Figure 2) [5,6,7,8]. Interestingly, the presence of a 9-O acetyl group in sialic acid can have an impact on the activity of sialidases, which remove sialic acid from larger molecules [9].

Sialic acid O-acetylation can be present both in proteins and lipids. Membrane-bound acetyl-transferase activity was found to be associated with the modification of endogenous glycoprotein-bound sialic acids, while soluble activity was linked to the modification of exogenous, non-glycosidically bound sialic acids. This finding was further extended to brain tissue from pigs and cows [10]. These first discoveries did not make the distinction between protein-bound and lipid-bound acetylated sialic acids. The first isolation of a ganglioside containing 9-O acetylation was obtained in a mouse brain, within trisialo-ganglioside [11] and tetrasialo-ganglioside [12] structures. This was followed by guinea pig kidney [13], bovine buttermilk [14,15], codfish brain [16,17], rat and equine erythrocytes [18,19], as well as less common species, such as feather starfish [20]. In rat erythrocytes, a combination of thin-layer chromatography, gas chromatography, and an enzymatic treatment with *Vibrio cholerae* sialidase could identify GD1a (GD1 of the “a” series, bearing 1 sialic acid on the galactose in position II; 0-, b- and c-series bearing 0, 2 and 3, respectively) (Table 1) and not GM1 as the main ganglioside containing this modification. In equine erythrocytes, NMR and fast atom bombardment mass spectrometry (FABMS) could identify 9-O-acetyl-GM3 (9-O-acGM3) [19]. In human tissue, an analysis in a normal thyroid gland resulted in the identification of a potential presence of 9-O acetyl gangliosides, defined as containing alkali-labile sialic acid [21]. Additionally, an antibody claimed to recognize 9-O acetylated GD3 (9-O-acGD3) was able to bind normal human melanocytes [22], and so did another one isolated from melanoma cells [23]. This newly detected form was characterized using NMR and FABMS and further found in other species and tissues, such as rainbow trout, where it accounts for 23% of total gangliosides [24,25]. Finally, an acetylated trisialylated form, 9-O-acGT2, was first identified in cod brain [16].

### 1.2. Chemical Structure and Interactions

In GD1a, the N-acetylated sialic acid is linked to the outer galactose residue [26]. Conformational studies have been performed through molecular dynamics modeling and NMR on 9-O-acGD1a, concluding that acetylation does not modify the overall conformation of the ganglioside [26]. Specific interaction with a purified IgG fraction from human serum was suggested by the same study. More recently, a study on GM3 indicated that neither 9-O-acetylation nor 9-N-acetylation induces significant conformational changes on dihedral angles or the secondary structure, those being limited to the sialic acid glycerol chain and confirming structural similarities between both forms [27].

Concerning the composition in terms of sphingoid bases and acyl chains, this varies among species and no particular association with 9-O acetylation can be inferred from the scarce data available. Studies made on bovine buttermilk O-acetylated gangliosides have revealed C18-sphingosine as the sphingoid base and C18:0, C22:0, C23:0 and C24:0 as the main fatty acyl chains [15]. In rainbow trout ovarian fluid, the structure differs, as it contains 4-sphingenine as a sphingoid base, and C24:1 among fatty acids [25]. In another fish, mullet milt, 9-O-acGM3 is the predominant, acetylated form, containing mostly C18:1/C16:0 fatty acids [28]. In feather starfish, C16 sphingosine is accompanied by C22:0 or C24:0 as the most common acyl chains in an N-acetylated form [20].

### 1.3. Enzyme Regulation

Sialic acid O-acetylation appears as a cell-specific and developmentally regulated process. This is based on tightly regulated activity of 9-O-acetyltransferases. Pioneering studies indicate that sialyltransferase action regulates the expression of O-acyltransferases [29]. The cloning of this sialyltransferase (sialate-O-acetyltransferase, CASD1) was an elusive task. In one of the attempts, an open reading frame corresponding to a truncated form of the GC Vitamin-D-binding protein (VDBP) was found specifically responsible for sialic acid 9-O-acetylation of glycoproteins, while a fusion protein between a bacterial tetracycline resistance gene repressor and a sequence of the P3 plasmid (Tetrfusion) was able to acetylate gangliosides [30]. An interesting observation is that the product of O-acetylation makes the sialic acid moiety resistant to sialidase [31], which could have functional implications. Additionally, the natural forms of acetylated GD3, a disialylated ganglioside, present the modification at the terminal sialic acid moiety, as compared to synthetic forms [32]. In another study, it was shown that O-acetyltransferases use preferentially di- and tri-sialogangliosides as substrates rather than mono-sialogangliosides [33]. Acetyltransferase activity on GD3 (9-O-acGD3) is unchanged by the endoplasmic reticulum-to-Golgi transfer stimulator brefeldin A, suggesting that the activity resides in the same Golgi compartment as GD3 synthase, which is not the case for 9-O-acGD2 synthesis [34]. This suggests different compartments and potentially different enzymes for GD3 and GD2 modification. Nevertheless, 9-O-acGD2 can be synthesized either from GD2 by acetylation or from 9-O-acGD3 by glycosylation. It must be noted that the biosynthesis of 9-O-acetylated gangliosides requires a transfer of the acetyl group from acetyl-CoA. The Acatn acetyl-CoA transporter was identified in mice as intervening in this process and being mainly expressed during embryogenesis [35].

The 9-O-acetylation of GD3 has been proposed to be induced in Chinese hamster ovary (CHO) cells by the stable expression of its precursor, GD3, through activation of the *Tis21* gene [36]. Moreover, when cells are incubated in the presence of exogenous GD3, cellular 9-O-acGD3 is detected after 6 h and a half-life of 24 h is observed, suggesting the induction of the biosynthetic enzymatic machinery. This process, also reported in human fibroblasts, is inhibited by blocking the clathrin-mediated internalization of GD3 [37]. Conversely, Tis21 does not seem to be involved in the upregulation of 9-O-acGD3 synthesis that occurs in a GM2/GD2 synthase knockout mouse model to compensate for the lack of complex gangliosides [38]. In this model, Vitamin D receptor and acetyl CoA transporter are not upregulated, suggesting an alternative mechanism of synthesis.

Reports on pharmacological agents exerting an impact on these synthesis reactions are scarce. In one of the few examples, it has been shown that salicylate leads to the deacetylation of gangliosides [39]. Additionally, cytidinmonophosphate-sialic acid and acetyl-CoA inhibit in vitro sialyl transferase activity [40].

In addition to enzyme activity, the regulation of enzyme expression must be considered. To date, no precise regulatory mechanisms for *CASD1* or *SIAE* expression based on experimental evidence have been published. Nevertheless, their promoters are defined in the Ensembl database and several transcription factor binding sites have been confirmed in numerous cell lines via ChIP-seq within the ENCODE project (Tables S1 and S2). In addition, both promoters contain a CpG island (108 CpG in the *CASD1* promoter and 50 CpG in the *SIAE* promoter) (Figure S1). Interestingly, *SIAE* mRNA transcriptional variant 2 sequence starts upstream from its CpG island location, maybe as part of a mechanism to avoid silencing by methylation. Although the regulatory landscape of these two genes currently remains unknown, according to the Protein Atlas, endocrine tissues present the highest *CASD1* mRNA expression, followed by the eye and digestive tract, while the protein has been found in high abundance also in the brain, pancreas, reproductive tissues, bone marrow and lymphoid tissues [https://www.proteinatlas.org/ENSG00000127995-CASD1/tissue (accessed on 3 May 2023)]. *SIAE* mRNA shows the highest expression level in the gastrointestinal tract, while the highest protein expression corresponds to the brain, endocrine tissue, urinary system, male tissues and bone marrow and lymphoid tissues [https://www.proteinatlas.org/ENSG00000110013-SIAE/tissue (accessed on 3 May 2023)].

### 1.4. Methodological Points

Early studies and many of the follow up works have been based on the detection of this type of modified ganglioside using monoclonal antibodies in combination with thin-layer chromatography (TLC) or immunohistochemistry (IHC). The so-called JONES, VIM-2 [41], 13A and 27A [42], UM4D4 [43], CDW60 [44], and MT6004 [45] antibodies have been shown to detect 9-O-acGD3, while the SGR37 monoclonal antibody distinctly detects the de-N-acetyl form of GD3 [46]. It must be pointed out, though, that targeting lipid antigens in IHC can be seriously impacted by the use of organic solvents for fixation and deparaffination, such as acetone and xylol, respectively. Special care must be taken, as an incorrect fixation protocol is likely to induce artifactual results [47].

The specific binding of influenza C virus has also been considered as the basis of detection methods. This microorganism presents a higher affinity for 9-O-ac and a lower affinity for 7-O-ac glycoconjugates [48,49], regardless of the nature of the core moiety (lipid or protein). Virus binding is also able to discriminate monoacetylated sialic acids from polyacetylated [48]. As a consequence, recombinant soluble influenza C hemagglutinin has been used to characterize 9-O-acetyl sialylation [50]. Other molecules recognizing 9-acetylated sialic acid and displaying a specificity for gangliosides are monocyte ficolins, highly conserved oligomeric lectins involved in innate immunity [51].

As explained above, chemical characterization has been mainly based on NMR and FABMS. Finally, the evaluation of sialyl transferase and SIAE enzymatic activities has added a functional dimension to some studies [52].

## 2. 9-O Acetylation of Gangliosides in Pathophysiology 

### 2.1. In Cell Physiology

#### 2.1.1. Embryogenesis

Human embryonic stem cells present a high abundance of 9-O-acGD3 that generally decreases alongside differentiation [53,54]. A particular type of cancer cell (NTERA-2, a human embryonic carcinoma line) has been used to study the ontogeny of glycolipids in association with cell differentiation during embryonic development. In this model, ganglio-series, including 9-O-ac forms, replaced globo-series (glycosphingolipids containing at least two neutral sugar residues and no sialic acid) when differentiation was induced with retinoic acid [55].

These molecules have been mainly studied in the context of nervous system development. In particular, the presence of 9-O-acGD3 has been shown in neuroepithelial precursor cells [56]. An antigen expressed during neural development was identified as 9-O-acGD3 [57]. In developing rat retina, the pattern of 9-O-acGD3 and that of its precursor GD3 were determined by the reactivity to several monoclonal antibodies (JONES, R24). The two patterns differed; in the case of the 9-O acetylated form, a rise was found between day E15 and postnatal day 2, with a pronounced drop between postnatal days 2 and 4 [58]. 9-O-acGD3 has also been found in primary cultures of both neurons and glia (reviewed in [59]). In freshly dissociated retinal cells, 9-O-acGD3 was found to be present on amacrine photoreceptors and in ganglion cells [58]. In a chick embryo, a monoclonal antibody (8A2) allowed detecting 9-O-ac gangliosides in the optic fiber layer of the central retina [60]. Another study based on monoclonal antibody staining and on sialidase sensitivity concluded that a 9-O-ac form of GT3 (ganglioside C series) was also increased in rat cerebral cortex at day 14 of gestation, then progressively decreased and was absent in adult rats [61], along with its 9-O-acGD3 counterpart [62].

In the developing rat nervous system, acetylated gangliosides have been associated with regions characterized by cell migration [63], such as the olfactory epithelium, where they are involved in the formation of the mature olfactory bulb [64] and the hippocampus [65]. They were detected in relation to the cell stream migrating from the lateral ventricle rostral subventricular zone to the olfactory bulb, suggesting a function in cell migration [66]. These gangliosides were also isolated from 10-day embryonic chicken brain [67]. Concerning their cellular function, there is evidence that 9-O acetylated gangliosides play a role in the extension of growth cones in neurites [68], along with a regulation of the microfilament and microtubular structure of their cytoskeleton, probably modulating cell motility [69]. The same authors found 9-O-acGD3 localized to contact points of neural growth cones, associated with beta-1-integrin and vinculin [70].

The functional relevance during embryogenesis of the 9-O acetylation of sialic acid was studied through the generation of a transgenic mouse model overexpressing the sialic acid-specific acetylesterase of influenza C virus under the control of the metallothionein promoter [71]. This resulted in an arrest of development at the 2-cell stage. Using the phenylethanolamine-N-methyltransferase promoter, the authors induced expression in the retina and adrenal gland, leading to an impaired morphology and function of these organs.

#### 2.1.2. Postnatal Nervous System

The nervous system is generally rich in gangliosides, including 9-O-acGD3. In a mouse model constitutively knocked out for GM2/GD2 synthase, the lack of complex gangliosides is compensated by an accumulation of the precursors, namely GM3 and GD3, in nervous tissue [72]. This accumulation also includes 9-O-acGD3, suggesting that this molecule can take over some of the functions of the absent glycosphingolipids [38]. In postnatal rat retina, a dorsal–ventral gradient of 9-O-acGD3 has been reported, an observation based on the JONES monoclonal antibody [58], as well as in the adult olfactory bulb, but at lower levels than in the developing nervous system [66]. In a chicken, 9-O acetylated gangliosides were no longer detected in the adult in the central optic fiber. In contrast, they would remain in the inner and outer plexiform layer, and in the outer nuclear layer [60]. Likewise, 9-O-ac gangliosides have been found to be absent in rat adult hippocampus [65]. In primary cell cultures from the retina, they are present in the retinal ganglion but not in Muller cells [60]. In the rat subventricular zone, the presence of 9-O-acGD3 has been demonstrated from neural stem and progenitor cells to the adult brain [73]. To add insight into the subcellular distribution of these molecules, in olfactory ensheathing glia from rats, 9-O-acGD3 has been identified in membrane rafts [74].

With respect to the potential function of these molecules in the nervous system, in cerebellar astroglia isolated from rats, JONES staining was found in the contact sites of migrating granule cells and in radial glia when cultured in the presence of neurons [63,75]. Another study suggested a role in the regulation of both neuronophilic and gliophilic migration [76]. Staining is also present in neurons and glia involved in the axonal regeneration of the sciatic nerve in adult rats [77], which is defective in GD3 synthase knockout mice [78]. The same antibody blocks migration in a dose-dependent manner, adding evidence to the participation of 9-O-acetyl gangliosides in granule cell migration [75,79] through a calcium-signaling mechanism involving PY2 receptors [80]. Anti-9-O-acGD3 antibody-based inhibition of olfactory ensheathing glia migration has been observed in organotypical cultures [81]; the inhibition of neuronal migration has been shown in vivo in normal mice [82,83] and confirmed through videomicroscopy [84], while migration was also blocked by a broad inhibitor of ganglioside synthesis (D-threo-1-phenyl-2-palmitoylamino-3-pyrrolidino-1-propanol, an inhibitor of the ganglioside precursor glucosylceramide) [84]. However, the fact that antibody-based inhibition also occurs in GD3 synthase knockout mice, which are not supposed to contain the acetylated derivative, suggests that the antibody inhibits migration through an alternative mechanism, while it also raises questions on its specificity [83]. Nevertheless, sciatic regeneration is perturbed in this mouse model and rescued by the administration of exogenous GD3, which supports a genuine role for downstream-generated gangliosides [78].

9-O-acetylated glycolipids have been detected in mammalian cerebellar Purkinje cells [85], where they occupy the rostral lobes in mice [86]. They mostly mark the late-onset sagittal banding patterns [87]. Interestingly, in the so-called nervous mutation model of mouse Purkinje cells, the surviving mutant cells in the cerebellum correspond to those positive for 9-O-acetylated gangliosides [86], mainly corresponding to 9-O-acGD3 [88].

#### 2.1.3. Immune System

Some glycolipid antigens at the surface of T lymphocytes were initially recognized by monoclonal antibodies and defined as CDw60. These molecules have been shown to induce costimulatory signals. The CDw60 antigen, also recognized by influenza C virus glycoprotein, was characterized as 9-O-acGD3 [89]. T lymphocytes (mostly CD4^+^) and granulocytes present high amounts of this CD60 antigen, in contrast to the low levels present in B cells, thymus cells and monocytes [90]. It was estimated that about 25% of peripheral T cells present a surface localization of CD60, while roughly all T cells express modest amounts intracellularly in Golgi vesicles [91]. In an early report, a subtype of CD8^+^ T cells, also expressing CD60—a so-called a T helper CD8^+^ CD60^+^ subset—was claimed to provide help to B cells, while CD8^+^ CD60^-^ suppressed B cell differentiation. Both populations produced IL-2 equally, but CD60^+^ would secrete more IL-4 and less interferon gamma [92]. In spite of the low levels initially reported, CD60 has been proposed as an activation marker of human B cells, as peripheral and tonsillar B cells become CD60^+^ when activated by phorbol esters [93]. It must be pointed out that another acetylated form of GD3, 7-O-acGD3, was also found in human leukocytes, recognized by a specific monoclonal antibody that induced cell proliferation [94]. T cell receptor (TCR) activation results in the decreased presence of detectable 9-O-acetyl sialic acid at the surface of T cells, but this is mostly due to decreased sialomucins, which also contain this residue, and not necessarily to gangliosides [50]. In peripheral blood mononuclear cells (PBMC), treatment with a monoclonal antibody targeting 9-O-acGD3, but not with another one against non-acetylated GD3, was able to induce phosphorylation of the spleen tyrosine kinase (Syk, p72), involved in T and B cell receptor signal transduction, resulting in phosphoinositide mobilization and cell proliferation [95].

Following subsequent studies, CD60 was subdivided into CD60a (GD3), CD60b (the O-acetylated form), and CD60c (the N-acetylated form) [96]. The CD60b form was found present in tonsillar B cells in the activated germinal center, colocalizing in lipid rafts with Syk and Lyn, in line with previous results [93,95]. Hence, B cells can be costimulated by anti-CD60b and anti-IgM/IL-4. Extrafollicular T cells also present with CD60b and can be costimulated with anti-CD60 and phytohemagglutinin (PHA). Conversely, anti-CD60c—recognizing the N-acetylated form—has been found to be sufficient to induce proliferation [96]. In a thorough study on the presence of the three CD60 forms during the differentiation of T cells and B cells, CD4^+^ cells showed the strongest, and CD8^+^ cells the weakest, presence of CD60b at the surface in thymocytes. Both T and B cells presented CD60b staining in a patchy fashion as compared to the other forms. Interestingly, subcellular distribution studies following biochemical methods showed 9-O-acGD3 mainly localized to non-raft microdomains in T cells and to raft microdomains in B cells [45].

#### 2.1.4. Hematopoiesis

In human bone marrow, erythroid progenitors are rich in 9-O-acGD3, but the molecule is progressively lost during maturation, becoming proapoptotic in mature erythrocytes [97]. The presence of 9-O-acGD3 in lymphoid and erythroid cells is reviewed in [98].

#### 2.1.5. Kidney

Cultured visceral glomerular epithelial cells, podocytes, contain the specific epitope 9-O-acGD3 recognized by several monoclonal antibodies, such as 13A and 27A. The latter could immunoprecipitate with a noncharacterized podocyte protein [42]. This epitope was found by the 27A antibody to colocalize in podocyte lipid rafts with nephrin, a protein present in the slit diaphragm, a structure responsible for the podocyte intercellular interaction and a main constituent of the glomerular filtration barrier. These seminal works indicate the importance of this modified ganglioside in the physiology and function of the glomerular barrier [99].

### 2.2. In Cell Pathology—Diseases

#### 2.2.1. Cancer

The 9-O-acetylation of gangliosides has been extensively associated with cancer, and even considered as a marker of cell and tissue growth [100]. Very early studies on melanoma cells found in extracts a thin-layer chromatography band comigrating with 9-O-acetylated gangliosides [101]. It was estimated that 10% of gangliosides in melanoma cells presented this modification. These modified sialic acids, independently of their associated moiety—either protein or sphingolipid—were recognized by a monoclonal antibody prepared against the rat brain tumor cell line B49. In another study, chromatographic comigration with GD3 was found in cell extracts after isolation with a monoclonal antibody derived from the immunization of mice with WM164 melanoma cells [23]. It was estimated that all nevus cell lines and one third of melanoma cell lines were positive to an antibody detecting this modification, which was also found in lymphocytes infiltrating 30% of tumors. Ever since, 9-O-acGD3 has been considered as a melanoma antigen [57,102,103,104], as has 9-O-acGD2 [105]. When evaluating different stages of Bomirski melanomas, 9-O-acGD3 was found increased in the amelanotic, fast-growing stage, as compared with the slow-growing, highly differentiated forms [106], suggesting a role for the molecule in cell growth. Its presence in nodular melanoma has been found to be greater than in metastatic acral lentiginous melanoma [107]. However, it has not been found present in uveal melanoma [108,109], which may indicate that the acetylated varieties are characteristic of metastatic forms (cutaneous) as compared with non-metastatic (uveal). Interestingly, while other gangliosides, such as GD2 and GD3, have been found to be increased in the serum of melanoma patients, this is not the case for 9-O-acGD3 [110].

In hamster melanoma, the O-acetylated form of GD3 was characterized as 7-O instead of the human 9-O. The structure of the former is not very different from that of buttermilk ganglioside, as it contains C18:0 sphingosine and a slightly different fatty acid composition: C16:0, C18:0, C20:0, C22:0 and C24:0 [111]. In human melanoma, a quite-high presence of C24:1 has been reported in both the 9-O-acGD3 and the GD3 precursor [23,112]. Melanoma cells also display de-N-acGD3 (resulting from the loss of the 5-N-acetyl group), with an intracellular and non-lysosomal distribution [46]. In this case, the main esterifying fatty acids are C16:0 and C18:0 [112].

In mouse erythroleukemia cells, 9-O-acGD3 is also present but not detectable at the surface, where 9-O-acetyl sialic acid is associated with sialomucins [113]. In lymphoblasts from acute lymphoblastic leukemia patients, 9-O-acGD3 levels are increased [114]. Increased SiAOAT enzymatic activity was detected in the microsomes of these cells. The activity was found to be higher at diagnosis and decreased in remission, whereas SIAE activity is down in the cytosol and in lysosomes [40,52]. In Sézary syndrome, a very aggressive leukemic form of cutaneous T cell lymphoma, circulating levels of CD60b (9-O-acGD3)-positive T cells were found to be associated with a poor prognosis [115].

9-O acGD3, along with other gangliosides, has been proposed as a marker of several neuroectodermal cancers. For example, it was detected in basal cell carcinoma cells and found to be dramatically increased as compared to normal epidermis or dermis [116,117]. It has been suggested as a marker of small-cell lung cancer [118]. Studies in breast tissue have demonstrated the presence of CD60 antigen in the Golgi apparatus of normal ductal cells, and increased in atypical hyperplasia and other benign lesions, as well as in mammary carcinoma cells [119]. In well-differentiated and invasive duct carcinoma, the antigen, identified as 9-O-acGD3, was found mostly present at the surface, with decreased presence in nondifferentiated carcinomas [119]. In some breast cancer cell lines (Hs 578T and SUM159PT), 9-O-acGD2 but not 9-O-acGD3 has been identified [120], and CASD1 has been demonstrated as the enzyme responsible for its synthesis [121]. Both GD3 and 9-O-acGD3 were detected and increased in 13 neural tumor cell lines [122] and in glioblastoma, where a critical ratio between the two forms promoting tumor survival was established [123]. As a consequence of all these findings, the presence of acetylated gangliosides in blood as cancer biomarkers has been considered and specific testing by liquid chromatography–mass spectrometry on dry blood samples has been developed [124].

The link between the 9-O-acetylation of gangliosides and cancer is underlined by its effect on apoptosis. GD3 is considered as a proapoptotic agent, at least in vitro, while its 9-O acetylated form is shown as antiapoptotic [39,125,126]. The presence of 9-O-acGD3 in Jurkat and Molt-4 cells prevents cell death induced by proapoptotic agents such as N-acetyl sphingosine and daunorubicin [39]. Lymphoblasts from lymphoblastic leukemia patients accumulate 9-O-acGD3 in mitochondrial membranes [114]. Unlike GD3, exogenous 9-O-acGD3 prevents mitochondrial membrane depolarization, cytochrome C release and caspase activation in lymphoblasts [114]. Interestingly, 9-O-acGD1, also known as neurostatin, has antiproliferative effects on astrocytoma cells [127] and synthetic forms have been produced and approved as anticancer drugs [128]. The potential regulation of apoptosis by acetylated gangliosides (CD60) has been addressed in lymphocytes [96]. However, a hematopoiesis study conducted on human bone marrow revealed a proapoptotic impact of 9-O-acGD3 on mature erythrocytes, in contrast to its effect on lymphoblasts [97].

9-O acetyl-GD3 was consequently proposed as a potential target for immunotherapy [129,130]. The antibody response to injection in melanoma patients of 9-O-acGD3 extracted from buttermilk was studied, but the reactivity was not found antigen specific [131], which underlies the problem of the low immunogenicity of the molecule. This was improved by combining the antigen with very-low-density lipoproteins and enhanced by IL-2, which could be used as adjuvants [132]. 9-N-acGD2, used as a stable surrogate of 9-O-acGD2, has been also used as antigen, in this case conjugated with the carrier bacteriophage Qbeta, eliciting a strong and long lasting immune response in dog [133]. Interestingly, a high titer of anti-9-O-acGD3 antibodies has been found in the serum of medulloblastoma patients [122]. Finally, in glioblastoma cells, several strategies based on hemagglutinin esterase cleavage of the acetyl group have been explored [123].

#### 2.2.2. Infection

Influenza C virus is known to infect cells through binding to N-acetyl-9-O-acetyl sialic acid, an ability that is shared with bovine coronavirus [134,135]. Treatment of cells with 9-O acetylesterase confers resistance to infection, which is reversed by treating cells with ganglioside preparations from bovine brain containing 9-O acetylated forms, suggesting 9-O-acetylated gangliosides as potential receptors for this pathogen [5]. Binding to 9-O-acGD1a has been demonstrated [136]. Conversely, influenza C virus is able to slowly hydrolyze in vitro 9-O-acGD1a [7] and 9-O-acGT3 [137], since the hemagglutinin encoded by the viral genome possesses a 9-O-acetyl sialic-acid-specific acetyl esterase activity [71]. Another pathogen, *Mycobacterium leprae*, invades Schwann cells with the help of endogenous 9-O-acGD3, which is also upregulated upon infection. Immunoblocking of the ganglioside reduces the demyelinization effect of the bacterium [138].

#### 2.2.3. Autoimmune Diseases

9-O-acGD1b has been associated with Guillain–Barré syndrome, an autoimmune disorder characterized by the presence of anti-glycolipid antibodies in the blood. The serum of a subset of patients reacts with this modified ganglioside, along with the non-acetylated form, and with GM1, as found using ELISA and thin-layer chromatography immunostaining [139].

Psoriatic basal and suprabasal keratinocytes express 9-O-acGD3 at the surface, and the extent of expression is increased when these cells are subjected to material secreted by T cells isolated from the same lesions, suggesting that soluble factors secreted by T cells are responsible for this effect. In the same context, IL-4 and IL-13 induced the upregulation and interferon gamma downregulation of the ganglioside, while the upregulation effect was reduced by an anti-IL-13 antibody [43].

#### 2.2.4. Toxicology

Lead exposure has been associated with increased detection of several gangliosides in kidney, including 9-O-acGD3 in glomeruli, using monoclonal antibodies and confirmed with thin-layer chromatography [44]. This was suggested by the authors of the work to constitute a marker of lead exposure and to be associated with a dysregulation of apoptosis, in that high levels of 9-O-acGD3 in glomeruli were correlated with a lower number of apoptotic cells in the kidney.

## 3. Concluding Remarks: From Controversy to Future Prospects

The fact that detection systems target the acetylated sialic acid moiety, present in both gangliosides and glycoproteins, leads to the ambiguous interpretation of many results in the absence of further biochemical characterization. Thus, a thorough study on the expression of CD60 antigen in T cells and melanoma cells led to the conclusion that it corresponds mostly to a glycoprotein marker in the former and a glycolipid in the latter [140]. Another example of this ambiguity is the reported recognition by the JONES antibody of β1-integrin in mouse cerebellum [83], which compromises some conclusions based on this particular tool. Considering these constraints, mass spectrometry emerges as the most reliable approach to search for the distribution and biological effects of 9-O-acetylated gangliosides.

Some points raised by previous works will need to be clarified, while others are as yet unexplored. For example, a basic question is the relationship between the cell cycle and 9-O-acetylated ganglioside synthesis. Another one is the subcellular distribution of these molecules. Previous studies have shown their presence in mitochondria, at the plasma membrane surface in and out of raft-like membrane microdomains; yet, to date, little is known about their function in these compartments. Conversely, their presence in the nucleus has not been explored.

Regarding the likely abundance of 9-O-ac gangliosides in membrane raft-like microdomains, a potential function as entry points to viral particles could be hypothesized. It has been shown that the sialic acid moieties of gangliosides, by means of their negative charge, determine the electrostatic potential and thereby impact the interaction of viruses, such as SARS-CoV-2 with host cells [141,142]. Interestingly, SARS-CoV-2 spike protein binds preferentially to 9-N-ac and 9-O-ac sialic acid [143]. It is tempting to hypothesize that the 9-O-acetylation of gangliosides changes the dynamics of virus–raft interaction and eventually virus entry. Whether this is the case and whether the mechanism involves a receptor-like or a change in electrostatic interaction remain to be clarified.

While a reasonable body of knowledge has been gathered for 9-O-acetylated gangliosides in the context of cancer, an aspect that has been insufficiently addressed is their implications in other pathologies, especially those accounting for alterations in lipid metabolism (i.e., cardiovascular disease, type 2 diabetes mellitus, and nonalcoholic fatty liver disease) or lipid storage disorders. Likewise, the presence of 9-O-acetylated gangliosides in circulating macromolecular structures, such as lipoproteins or extracellular vesicles, is currently unexplored (apart from the enhanced immunogenicity of 9-O-acGD3 when adsorbed onto very-low-density lipoproteins [132]).

Finally, in light of the available data summarized in this review (Table 2), a question arises on the levels of 9-O-ac gangliosides found in physiological and pathological conditions. As suggested by several studies, these molecules play a key role in cell survival and cell mobility. These two properties are relevant to cancer cells to avoid immune defense mechanisms and to propagate throughout the body. This would explain why some 9-O-ac gangliosides are overabundant in cancer cells, hereby displaying potential as cancer biomarkers. Nevertheless, these roles are also important in other cells in physiological conditions. Consequently, 9-O-ac gangliosides are not exclusive to cancer cells and their role as cancer biomarkers can be contested. For example, melanocytes increase their 9-O-acGD3 content during carcinogenesis. However, other cells in physiological conditions (e.g., podocytes, neuroblast cells, and lymphocytes) have been proven to contain the same molecule, which somehow represents a paradox. It can be hypothesized that their physiological/pathological role in cells depends on a combination of at least two parameters, namely abundance (as shown in [104]) and subcellular location. An additional parameter would be the ratio between 9-O-ac and nonacetylated counterparts [39,125,126], or between different types of acetylated forms (i.e., 9-O-ac, 7-O-ac, and N-ac). Even the fatty acyl chain esterifying the ceramide moiety could play a part [20,25,28]. This requires a global analysis of all ganglioside forms, and further underlines the importance of mass-spectrometry-based methods.

In conclusion, the results so far point towards a relevant role of 9-O-ac gangliosides in many tissues and cellular mechanisms. Nevertheless, the available information is highly fragmented and further systematic research will be necessary to pursue the understanding of this fascinating puzzle.

## Figures and Tables

**Table 1:** Main structural characteristics of the gangliosides cited in the text.

Acronym	Sialic Acid Modification	Main Structural Features
GM	N/A	One sialic acid residue
GD	N/A	Two sialic acid residues
GT	N/A	Three sialic acid residues
GM1	N/A	One sialic acid and four neutral sugar residues
GD1	N/A	Two sialic acid and four neutral sugar residues
GT3	N/A	Three sialic acid and two neutral sugar residues. All three sialic acid residues are linked to galactose residue in position 2 from the ceramide backbone.
9-O-acGM3	O-acetylated sialic acid	One sialic acid and two neutral sugar residues; O-acetylation on carbon 9 of one sialic acid.
9-O-acGD3	O-acetylated sialic acid	Two sialic acid and two neutral sugar residues; O-acetylation on carbon 9 of one sialic acid.
7-O-acGD3	O-acetylated sialic acid	Two sialic acid and two neutral sugar residues; O-acetylation on carbon 7 of one sialic acid residue.
9-N-acGD2	N-acetylated sialic acid	Two sialic acid and three neutral sugar residues; N-acetylation on carbon 9 of one sialic acid residue.
9-O-acGD1a	O-acetylated sialic acid	Two sialic acid and four neutral sugar residues; O-acetylation on carbon 9 of one sialic acid residue. One sialic acid residue is linked to the galactose as second neutral sugar from the ceramide backbone.
9-O-acGD1b	O-acetylated sialic acid	Two sialic acid and four neutral sugar residues; O-acetylation on carbon 9 of one sialic acid residue. The two sialic acid residues are linked to the galactose as the second neutral sugar from the ceramide backbone.
9-O-acGT2	O-acetylated sialic acid	Three sialic acid and three neutral sugar residues; O-acetylation on carbon 9 of one sialic acid residue. The three sialic acid residues are linked to the galactose as the second neutral sugar from the ceramide backbone.
9-O-acGT3	O-acetylated sialic acid	Three sialic acid and two neutral sugar residues; O-acetylation on carbon 9 of one sialic acid residue. The three sialic acid residues are linked to the galactose as the second neutral sugar from the ceramide backbone.

**Table 2:** Synthesis of reported observations involving different 9-O-acetyl gangliosides in physiological and pathological conditions.

Embryogenesis			
Date (Reference)	Observation	Sample	Detection Method: Target Molecule
1987 [55]	Ganglio-series replace globo-series when differentiation is induced by retinoic acid	NTERA-2 (human embryonic carcinoma line)	TLC + Antibody (ME-311): 9-O-acGD3
2005 [56]	9-O-acGD3 presence in neuroepithelial precursor cells	Neuroepithelial precursor cells	FC + Antibody (D1.1): 9-O-acGD3
1988 [58]	9-O-acGD3 rise between day E15 and postnatal day 2, and pronounced drop between day 2 and day 4 PN	Rat developing retinae	IF + Antibody (JONES): 9-O-acGD3
1991 [60]	Detection of 9-O-ac gangliosides in the optic fiber layer of central retina	Cultured cells from chicken embryo retinae	TLC/electron microscopy + Antibody (Mabs D1.1/JONES and 8A2): 9-O-acGD3 and unspecific gangliosides
1989 [61]	9-O-acGT3 increased in rat cerebral cortex at day 14 of gestation, then decreased and was absent in adult rats	Fetal rat cerebral cortex	TLC + Antibody (M6704): c-series gangliosides.
1997 [62]	9-O-acGD3 increased in rat cerebral cortex at day 14 of gestation, then decreased and was absent in adult rats	Fetal rat cerebral cortex	TLC + Antibody (493D4): O-acGD3, O-acLD1, O-acGD2 and O-acGD1b
1990 [63]	Acetylated gangliosides associated with granule cell migration (neurons) and glial cells require some form of neuron-glia interaction to display acetylated gangliosides	Cultured cells from 2 to 6 day postnatal rat cerebellums	ICC + Antibody (JONES): JONES antigens
1994 [64]	Acetylated gangliosides associated with the formation of mature olfactory bulb	Developing embryonic rat nervous system and postnatal rats	IHC + Antibody (JONES): JONES antigens
1996 [65]	Acetylated gangliosides associated with the formation of hippocampus and rapid decrease after birth.	Embryonic, postnatal and adult rat hippocampus	IHC + Antibody (JONES): JONES antigens
1996 [66]	9-O-ac gangliosides are involved in tangential cell migration both in lateral ventricle and rostral subventricular zone, along the rostral migratory stream and in the olfactory bulb in developing animals and, at lower levels, in adulthood.	Embryonic, postnatal and adult rat brain	IHC + Antibody (JONES): JONES antigens
1990 [67]	Monoclonal antibody A2B5 detects GT3, 9-O-acGT3 and other antigens. All A2B5-detected antigens decrease during chicken brain development	10-day embryonic chicken brain	TLC + Antibody (A2B5): GT3 and 9-O-acGT3
1996 [68]	9-O-ac gangliosides play a role in the extension of growth cones in neurites	Neurons of embryonic rat dorsal root ganglia explants grown on laminin substratum	IHC + Antibody (JONES): JONES antigens
1997 [69]	9-O-ac gangliosides regulate the microfilament and microtubular structure of neurites	Unavailable information	Unavailable information
2003 [70]	9-O-acGD3 localizes in contact points of neural growth cones and is associated with β-1-integrin and vinculin	Cultured neurites from dorsal root ganglia from embryonic rat	IHC + CM + Antibody (JONES): JONES antigens
1991 [71]	The cleavage of 9-O-ac esters on sialic acids causes 2-cell stage arrest in murine embryogenesis	Transgenic mice with a loss of O-ac of Sialic Acid	N/A
1991 [71]	Cleavage of 9-O-ac esters on sialic acids in retina and adrenal gland leads to impaired morphology and function in these organs (postnatal)	Transgenic mice with a loss of O-ac of sialic acid in adrenal gland and retina	IHC + Antibody(27A): 9-O-acGD3
**Postnatal nervous system**			
**Date (Reference)**	**Observation**	**Sample**	**Detection Method: Target Molecule**
2008 [38]	The absence of GM2/GD2 in nervous tissue increases GM3 and GD3 (this also includes 9-O-acGD3)	GM2/GD2 synthase KO mice	TLC + Antibody (JONES and GMR2): 9-O-acGD3
1988 [58]	Dorsal–ventral gradient of 9-O-acGD3 in postnatal rat retina	Developing rat retina	IHC + ICC + Antibody (JONES and R24)
1996 [66]	Dorsal–ventral gradient of 9-O-acGD3 in lateral ventricle rostral subventricular zone, along the rostral migratory stream and in the olfactory bulb at lower levels than in the developing nervous	Embryonic, postnatal, and adult rat brains	IHC + Antibody (JONES): JONES antigens
1991 [60]	9-O-ac gangliosides are not detected in the central optic fiber. In contrast, they remain in the inner and outer plexiform layer, and in the outer nuclear layer,	Adult chicken	TLC/electron microscopy + Antibody (Mabs D1.1/JONES and 8A2): 9-O-acGD3 and unspecific gangliosides
1996 [65]	9-O-ac ganglioside is absent in rat adult hippocampus	Adult rat	IHC + Antibody (JONES): JONES antigens
2017 [73]	9-O-acGD3 presence in subventricular zone from neural stem and progenitor cells in the adult	Postnatal Lister Hooded rats	IHC + Antibodies (CD60b and JONES): CD60b antigens
1990 [63]	Acetylated gangliosides associated with granule cell migration (neurons) and glial cells require some form of neuron-glia interaction to be displayed	Cultured cells from 2 to 6 day postnatal rat cerebellums	ICC + Antibody (JONES): JONES antigens
2001 [75]	Finding of 9-O-acGD3 in the contact sites of migrating granule cells and in radial glia; 9-O-acGD3 involvement in granule cell migration in the developing cerebellum	Postnatal rat cerebellum and rat cerebellar explants	IHC/IF/IEM + Antibody (JONES): JONES antigens.
2001 [74]	The identification of 9-O-acGD3 in membrane rafts	Primary culture of olfactory ensheathing glia from rat	Membrane raft isolation. Dot blotting + Antibody (JONES)
2001 [76]	9-O-acGD3 may participate in neuronophilic and gliophilic migration	Culture explants of anterior subventricular zone (SVZ) of cerebral cortex from postnatal rats	CM + Antibody (JONES): JONES antigens Immunoblockage (JONES)
2007 [77]	9-O-acGD3 is re-expressed in neurons and glia cells involved in axonal regeneration	Sciatic nerve from adult rats and its explant culture	CM + Antibodies (mouse IgM monoclonal anti-9-O-acGD3 (Sigma) and JONES): 9-O-acGD3
2014 [78]	Defective axonal regeneration in GD3 synthase KO that can be rescued through administration of exogenous GD3	Sciatic nerve from adult rats and its explant culture	N/A
2005 [79]	Participation of 9-O-ac gangliosides in granule cell migration	Neuron-like cultured cells derived from P19 embryonal carcinoma stem cells	TLC/IF + Antibodies (Jones and D1.1): 9-O-acGD3. Blockage of migration (JONES)
2012 [80]	Participation of 9-O-ac gangliosides in granule cell migration through a calcium-signaling mechanism involving PY2 receptors	Explant culture from mouse early postnatal cerebellum	IF + Antibody (JONES). Blockage of migration (JONES)
2019 [81]	Antibody inhibition of olfactory ensheathing glia migration	Organotypical olfactory ensheathing cultures from rats	IF+ anti-9-O-acGD3 (mouse IgM monoclonal antibody; Sigma). Immunoblockage (JONES)
2004 [82]	Inmunoblockage of neuronal migration by JONES antibody but not by A2B5 antibody	Cerebellar granule neurons from postnatal rats	CM + BrU. Immunoblockage (JONES)
2007 [83]	Independence of the mice model in the inhibition of neuronal migration by JONES antibody + JONES-positive proteins raises questions on antibody specificity	Cerebella from wild-type and GD synthase KO mice	IHC, IF, TLC, WB + Antibodies (JONES, D1.1, or A2B5 (c-series gangliosides))
2012 [84]	Inhibition of neuronal migration by inmunoblocking with JONES antibody; 9-O-acGD3 role in cell–cell and cell–substrate interactions in neuroblast	Subventricular zone explants from rat brain	Videomicroscopy, IF, Immunoblockage (JONES)
1992 [85]	Two subtypes of Purkinje cells contain 9-O-ac glycolipids	Adult mice cerebellum	IHC/TLC + Antibody (P-path): 9-O-ac glycolipids
1994 [86]	Nervous mutation-surviving Purkinje cells in the cerebellum correspond to those positive for 9-O-ac gangliosides	Nervous mutation (nr/nr and nr/+) and wild-type (+/+) mice	IHC + Antibodies (P-path): 9-O-ac glycolipids (9-O- acGD3 and 9-O-acLDI)
1999 [87]	Purkinje cell P-path antigens mark the late-onset sagittal banding patterns and they are En-2-sensitive	Postnatal wild-type and En-2 mutant mice	IHC + Antibodies (P-path): 9-O-ac glycolipids (9-O- acGD3 and 9-O-acGD1)
1994 [88]	Nervous mutation-surviving Purkinje cells in the cerebellum correspond to those positive mainly for 9-O-acGD3	Nervous mutation (nr/nr and nr/+) and wild type (+/+) mice	IHC/TLC + Antibody (P-path): 9-O-ac glycolipids (9-O- acGD3 and 9-O-acLDI)
**Immune system**			
**Date (Reference)**	**Observation**	**Sample**	**Detection Method: Target Molecule**
1994 [89]	Characterization of T lymphocyte CDw60 antigen as 9-O-acGD3	Leukocytes from children’s tonsils and from healthy adult donors	TLC + influenza C virus incubation: 9-O-ac gangliosides
1995 [90]	T lymphocytes (mostly CD4^+^) and granulocytes present high amounts of CD60 antigen, in contrast to low levels present in B cells, thymus cells and monocytes	Human leukocytes	TLC + Antibodies R24 do not detect 9-O-acGD3 but UM4D4 does (unspecific). Mass spectrometry
2000 [91]	25% of peripheral T cells present a surface localization of CD60, while roughly all T cells express intracellularly CD60 in Golgi vesicles	T lymphocytes	FC/IEM + Antibody (M-T32): CD60 antigen
1994 [92]	CD8^+^ CD60^+^ subset of T cells (T helper CD8^+^) secretes more IL-4 and less interferon gamma than CD8^+^ CD60^-^ T cells	T lymphocytes from healthy volunteer donors	FC + Ab mAb M-T32: CD60 antigen
1997 [93]	CD60 is an activation marker of human B cells. Peripheral and tonsillar B cells become CD60^+^ when activated by phorbol esters	Peripheral blood lymphocytes from healthy donors and tonsillar B cells from children	FC/TLC + Antibodies (UM4D4, F6 and Z17): CD60
1997 [50]	TCR activation decreases 9-O-ac sialic acid at the surface of T cells, but due to decreased sialomucins, not necessarily to gangliosides	Mouse lymphocytes from either spleen or lymph nodes	Lipid extraction + ELISA (CHE-FcD): 9-O-ac sialic acid
1998 [95]	Induction of Syk, phosphoinositide mobilization and cell proliferation in PBMC by treatment with a monoclonal antibody targeting 9-O-acGD3	Human PBMC	TLC/FC/IEM + Antibodies (27A and R24): 9-O-acGD3 and GD3 respectively
2006 [96]	CD60 antigen is subdivided into CD60a (GD3), CD60b (9-O-acetylated form), and CD60c (7-O-acetylated form) Anti-CD60b with IL-4 can costimulate B cells CD60b is present in Extrafollicular T cells and can be costimulated with antiCD60b and PHA	Human tonsillar lymphocytes	IHC/CM/FC + Antibodies (R24, UM4D4 and U5): GD3, 9-O-acGD3 and 7-O-acGD3
	CD60b is present in tonsillar B cells in the activated germinal center, colocalizing in lipid rafts with Syk and Lyn		
2011 [45]	Both T and B cells present CD60b staining in a patchy fashion as compared to the other forms of CD60 antigen CD4^+^ cells show the strongest, and CD8^+^ the weakest, presence of CD60b at the surface in thymocytes Subcellular distribution of 9-O-acGD3 is non-raft microdomains in T cells and raft microdomains in B cells	Human tonsillar lymphocytes	IHC/CM/FC + Antibodies (R24, UM4D4 and U5): GD3, 9-O-acGD3 and 7-O-acGD3
**Hematopoiesis**			
**Date (Reference)**	**Observation**	**Sample**	**Detection Method: Target Molecule**
2007 [97]	9-O-acGD3 is present in human bone marrow erythroid progenitors, is progressively lost during maturation, and becomes proapoptotic in mature erythrocytes	Bone marrow and peripheral blood erythrocytes from children with acute lymphoblastic leukemia and clinical remission	FC + Antibody (JONES): 9-O-acGD3
**Kidney**			
**Date (Reference)**	**Observation**	**Sample**	**Detection Method: Target Molecule**
1996 [42]	Cultured podocytes contain 9-O-acGD3 and it immunoprecipitates with a non-characterized podocyte protein	Cultured podocyte line from rat glomerular explants	IF/IP + Antibodies (27A): 9-O-acGD3
2001 [99]	9-O-acGD3 colocalizes in podocyte lipid rafts with nephrin at the slit diaphragm, a constituent of the glomerular filtration barrier	Rat kidneys and glomeruli	IHC/IP/IEM + Antibody (27A): 9-O-acGD3
**Cancer**			
**Date (Reference)**	**Observation**	**Sample**	**Detection Method: Target Molecule**
2002 [100]	9-O-acetylation of gangliosides as a marker of cell and tissue growth in cancer	Review article	Review article
1984 [101]	Band comigrating with 9-O-ac gangliosides from melanoma cell lipid extracts	Rat (B49) and human (M14) melanoma cell lines	TLC + Antibody (D1.1): 9-O-acGD3
1985 [23]	9-O-ac gangliosides detected in nevi and melanoma cells and also in lymphocytes in 30% of cases studied	27 melanoma cell lines	FAB-MS + NMR/IHC + Antibody (ME 311)
1987 [102], 1989 [103], 1993 [104]	9-O-acGD3 considered as a melanoma antigen	20 melanoma cell lines and 5 human tissues	TLC + Antibody (D1.l): 9-O-acGD3
1992 [105]	9-O-acGD2 is a melanoma antigen	M21 melanoma cell line	FABS-MS/NMR/TLC + Antibodies(14.G2A): 9-O-acGD2
1989 [106]	9-O-acGD3 increased in amelanotic, fast-growing stage, as compared with slow-growing, highly differentiated forms, suggesting a role in cell growth	Hamster melanoma cells: Ab amelanotic melanoma (fast-growing), Ma melanotic melanoma (slow-growing), and MI hypomelanotic melanoma (slow-growing)	Unavailable information
1991 [107]	9-O-acGD3 presence in nodular melanoma higher than in metastatic acral lentiginous melanoma	Primary and metastatic acral lentiginous melanoma and nodular melanoma lesions from patients	Unavailable information
1989 [108], 1992 [109]	9-O-acGD3 is not present in uveal melanoma	Surgically removed uveal melanoma lesion	ME311 [108], TLC [109]: 9-O-acGD3
2007 [112]	In human melanoma, a high presence of sphingosine C24:1 in both 9-O-acGD3 and GD3	Human melanoma tumors	HPLC-GLC-MS/TLC: 9-O-acGD3, GD3
1996 [113]	9-O-acGD3 is present in mouse erythroleukemia cells intracellularly	Murine erythroleukemia (MEL) cells	Ganglioside extraction + ELISA (CHE-FcD, 27A): 9-O-ac gangliosides, 9-O-acGD3
2008 [114]	Lymphoblasts from acute lymphoblastic leukemia patients have increased levels of 9-O-acGD3 and it accumulates in mitochondrial membrane Exogenous 9-O-acGD3 (but not GD3) prevents mitochondrial membrane depolarization, cytochrome C release and caspase activation in lymphoblasts	(MOLT-4) ALL cell line and PBMC from patient	IEM/TLC +Antibody (MT-6004): 9-O-acGD3
2010 [115]	In Sézary syndrome, circulating levels of 9-O-acGD3-positive T cells are a malignancy marker	Human PBMC	FC + Antibody (anti-CD60 from BD Biosciences): 9-O-acGD3
1992 [116]	9-O-acGD3 is a marker of neuroectodermal cancers	Human skin from donors and nodular and sclerosis basal cell carcinoma from patients	TLC+ Antibody (JONES): 9-O-ac sialic acid
2001 [117]	9-O-acGD3 is increased in basal cell carcinoma cells	Human basal cell carcinoma tumor samples and healthy skin from patients and healthy donors	TLC + (influenza C virus and Antibody): MoAb against 9-O-acGD3
1997 [118]	9-O-acGD3 is a marker of small cell lung cancer	Small cell and non-small cell lung cancer cell lines	Antibody (limited information)
1998 [119]	In well-differentiated and invasive duct carcinoma, 9-O-acGD3 is present at the surface, with a decreased presence in non-differentiated carcinomas	Benign and malignant breast lesions and normal mammary gland tissue, cell lines of breast carcinoma (MCF-7 and EFM-19)	IHC/TLC + Antibody (M-T21): 9-O-acGD3
2019 [120]	In some breast cancer cell lines, 9-O-acGD2 and not 9-O-acGD3 has been identified	Breast cancer cell lines (Hs 578T, SUM159PT, MDA-MB-231 and MCF-7)	LCMS/FC/CM/IHC + Antibodies (7H2 mouse IgG3 and 8B6 mouse IgG3): anti-O-ac-GD3 and anti-O-acGD2, respectively.
2021 [121]	CASD1 is the enzyme responsible for 9-O-acGD2 as well as for 9-O-acGD3 synthesis	SUM159PT and CHO cell lines	TLC/IHC/CM + Antibodies (M-T6004 and 8B6): 9-O-acGD3 and O-acGD2, respectively.
2008 [122]	GD3 and 9-O-acGD3 increased in neural tumor cell lines High titer of anti-9-O-acGD3 antibodies in medulloblastoma patients’ serum	13 neural tumor cell lines + NSC-34, CHO cells, and fibroblasts as controls Sera from patients with neural tumors and healthy controls	TLC/ELISA + Antibodies (R24 and D.1.1): GD3 and 9-O-ac-GD3
2011 [123]	The ratio between GD3 and 9-O-acGD3 is critical to tumor survival in glioblastoma	Three glioblastoma cell lines: SNB-19, an in-house-derived adult biopsy cell line, and IN699	FC + Antibody (MB3.6 and Clone D1.1): GD3 and 9-O-acGD3
2002 [125], 2006 [39], 2014 [126]	GD3 is considered proapoptotic in vitro, while its 9-O-ac form is antiapoptotic	HEK-293 and U87 cells Jurkat and Molt-4 cell lines	FC/CM/TLC + Antibody (M-T6004, P-Path, UM4D4): 9-O-acGD3
2006 [39]	9-O-acGD3 in Jurkat and Molt-4 cells prevents cell death by proapoptotic agents (N-acetyl sphingosine and daunorubicin)	Jurkat and Molt-4 cell lines	FC/CM/TLC + Antibody (M-T6004): 9-O-acGD3
2004 [127]	9-O-acGD1 has antiproliferative effects on astrocytoma cells	Human glioma cell lines U-373 and T98G	N/A
2006 [96]	In lymphocytes, acetylated gangliosides (CD60) decrease apoptosis	Human tonsillar lymphocytes	IHC/CM/FC + Antibodies (R24, UM4D4 and U5): GD3, 9-O-acGD3 and 7-O-acGD3.
2007 [97]	Proapoptotic impact of 9-O-acGD3 on mature erythrocytes	Bone marrow and peripheral blood erythrocytes from children with acute lymphoblastic leukemia and clinical remission	FC + Antibody (JONES): 9-O-acGD3
1995 [129], 1997 [130]	9-O-acGD3 as a potential target for cancer immunotherapy	14 tumor cell lines: 7 melanomas, 3 neuroblastomas, 1 astrocytoma and 3 sarcomas	FC + Antibody (D1.1 and 5BI): 9-O-acGD3
1995 [131]	Antibody response in melanoma patients after injection of 9-O-acGD3 not antigen-specific	N/A	N/A
1997 [132]	Improved antibody response in mice after injection of 9-O-acGD3 combined with VLDL and enhanced by IL-2	BALBc mice	ELISA/TLC + Antibody (MAb 7H2)
2021 [133]	9-N-acGD2 (9-O-acGD2 surrogate) conjugated with a carrier bacteriophage (Qbeta) elicits a strong and long-lasting immune response	dogs	N/A
**Infection**			
**Date (Reference)**	**Observation**	**Sample**	**Detection Method: Target Molecule**
1996 [134]	Influenza C virus infects cells through binding to N-acetyl-9-O-ac sialic acid, like bovine coronavirus	Polarized Madin–Darby canine kidney (MDCK) cells	N/A
2021 [135]	Human CoVs OC43 and HKU1, and human orthomyxovirus ICV, preferentially bind to 9-O-ac α2,8-linked sialosides	HEK-293T cells	N/A
1987 [5]	Treatment of cells with 9-O acetylesterase confers resistance to influenza C virus infection; this is reversed by ganglioside containing 9-O-ac forms	MDCK II cells	N/A
1992 [136]	Influenza C virus binds to 9-O-acGD1a	Immobilized glycoconjugates	TLC: 9-O-acGD1a
1988 [7]	Influenza C virus is able to hydrolyze in vitro 9-O-acGD1a	N/A	TLC/LC-MS: GD1a
1991 [137]	Influenza C virus is able to hydrolyze in vitro 9-O-acGT3	N/A	TLC + Antibody (A2B5): GT3
1991 [71]	Influenza C virus hemagglutinin contains a 9-O-ac sialic-acid-specific acetyl esterase activity	Transgenic mice with partial or total loss of O-acetylation of sialic acids	IHC + Antibody (27A): 9-O- acGD3
2010 [138]	Mycobacterium leprae invades Schwann cells with the help of endogenous 9-O-acGD3; immunoblocking of the ganglioside reduces the demyelinization effect of the bacterium	Schwan cell line (ST-8814) and mice	CM/TLC + Antibody (JONES): 9-O-acGD3. Inmunoblockage
**Autoimmune disease**			
**Date (Reference)**	**Observation**	**Sample**	**Detection Method: Target Molecule**
1996 [139]	The serum of some Guillain–Barré syndrome patients reacts with 9-O-acGD1b, GD1b and GM1	Patients’ serum	ELISA
1997 [43]	Psoriatic basal and suprabasal keratinocytes display 9-O-acGD3 at the surface This is upregulated by IL-4 and IL-13, and dowregulated by IFNγ secreted by T cells	Primary cultures of keratinocytes and biopsies	FC/IHC + Antibody (UM4D4): 9-O-acGD3
**Toxicology**			
**Date (Reference)**	**Observation**	**Sample**	**Detection Method: Target Molecule**
2008 [44]	Association between lead exposure and an accumulation of 9-O-acGD3 and other gangliosides in glomeruli	Male Wistar rat kidneys	IHC/TLC + Antibody (CDW60): 9-O-acGD3

CHE-FcD = Hemagglutinin Esterase of Influenzavirus C fused to the carboxyl end with human IgG1 Fc region treated with diisopropylfluorophosphate to eradicate its esterase activity. CM: Confocal microscopy. FAB-MS: Fast atom bombardment mass spectrometry. IEM: Immunoelectron microscopy. IF: Immunofluorescence. IHC: Immunohistochemistry. IP: Immunoprecipitation. TLC: Thin-layer chromatography.

## Data Availability

The data contained in the supplementary material have been obtained from the Ensembl database (https://www.ensembl.org/Homo_sapiens/Regulation/Summary?db=core;fdb=funcgen;g=ENSG00000127995;r=7:94509219-94557019;rf=ENSR00000215297; https://www.ensembl.org/Homo_sapiens/Regulation/Summary?db=core;fdb=funcgen;g=ENSG00000110013;r=11:124633113-124695707;rf=ENSR00000046429); the UCSC genome database (http://genome.ucsc.edu/cgi-bin/hgTracks?db=hg19&lastVirtModeType=default&lastVirtModeExtraState=&virtModeType=default&virtMode=0&nonVirtPosition=&position=chr7%3A94139170%2D94186328&hgsid=1620065055_IR0bazPQThDkcqjiqyjTZvZZi1oC); and the Human Protein Atlas (https://www.proteinatlas.org/ENSG00000127995-CASD1/tissue; https://www.proteinatlas.org/ENSG00000110013-SIAE/tissue).

## Supplementary Materials

The following supporting information can be downloaded at: https://www.mdpi.com/article/10.3390/biom13050827/s1, Figure S1: Alignment of CASD1 and SIAE (B) NCBI transcript variants to human genome and its CpG Islands; Table S1: CASD1 promoter transcription factor binding sites; Table S2: SIAE promoter transcription factor binding sites.

## Abbreviations

7-O-ac:	7-O-acetylated
9-N-ac:	9-N-acetylated
9-O-ac:	9-O-acetylated
9-O-acLD1:	disialosyl-lacto-N-neotetraosylceramide (LD1)
CASD1:	CAS1 domain containing
CDw60 = CD60:	9-O-acetylated GD3 antigen
CD60a:	GD3 (non-acetylated) antigen
CD60b:	9-O-acGD3 antigen
CD60c:	7-O-acGD3 antigen
CHE-FcD = hemagglutinin Esterase of Influenzavirus C fused to the carboxyl end with human IgG1 Fc region treated with diisopropylfluorophosphate to eradicate its esterase activity.	
CM:	confocal microscopy
FABMS:	fast atom bombardment mass spectrometry
IEM:	immunoelectron Microscopy.
IF:	Immunofluorescence
IHC:	Immunohistochemistry
IP:	Immunoprecipitation
N-ac:	N-acetylation
NMR:	nuclear magnetic resonance
PHA:	Phytohemagglutinin
SIAE:	sialate O-acetylesterase
SiAOAT:	sialate O-acetyltransferase
TLC:	thin-layer chromatography
