# Supplementary material for: 9-O Acetylated Gangliosides in Health and Disease
Source: Biomolecules. 2023 May 12;13(5):827. doi: 10.3390/biom13050827 (PMC10216186; doi:10.3390/biom13050827)
Supplement: Supplementary file 1 [file biomolecules-13-00827-s001.zip › biomolecules-2372012-supplementary.pdf]

Figure S1. Alignment of CASD1 (A) and SIAE (B) NCBI transcript variants to human genome (GRCh37/hg19)* and its CpG Islands using USCS genome **

A) chr7:94,139,170-94,186,328

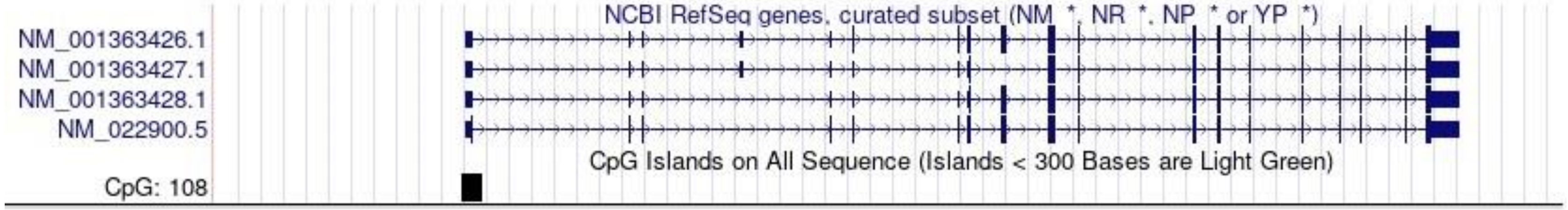

B) chr11:124,511,492-124,552,118

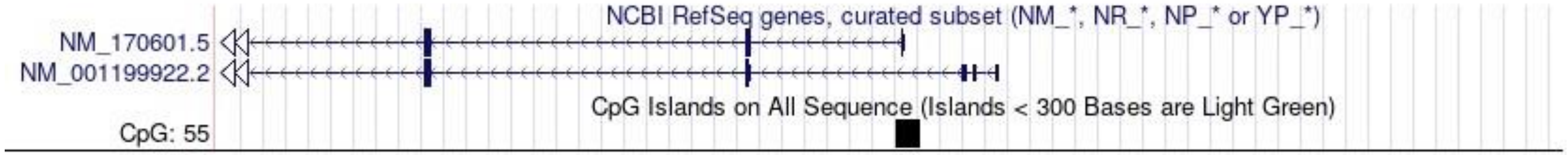

*Ensembl database : (2 may 2023), https://www.ensembl.org/Homo_sapiens/Regulation/Summary?db=core;fdb=funcgen;g=ENSG00000127995;r=7:94509219-94557019;rf=ENSR00000215297
https://www.ensembl.org/Homo_sapiens/Regulation/Summary?db=core;fdb=funcgen;g=ENSG00000110013;r=11:124633113-124695707;rf=ENSR0000004642
**UCSC Genome: (3 May 2023), http://genome.ucsc.edu/cgi-bin/hgTracks?db=hg19&lastVirtModeType=default&lastVirtModeExtraState=&virtModeType=default&virtMode=0&nonVirtPosition=&position=chr7%3A94139170%2D94186328&hgsid=1620065055_IR0bazPQThDkcqjiqyjTZvZZi1oC

Table S1. CASD1 promoter (ENSR00000215297) transcription factor binding sites (TFBS). CASD1 promoter (ENSR00000215297) TFBS deposited in ENCODE database according to ENSEMBL and filtering by Open chromatin & TFBS as Evidence Type.

Transcription Factor	Cell type	Transcription Factor	Cell type
AFF1	K562	MYC	A549, GM12878, H1-hESC_3, HUVEC, HeLa-S3, HepG2, K562, MCF-7
ARID3A	GM12878, HepG2, K562, MCF-7	MYNN	K562
ARNT	GM12878, HepG2, K562	Nanog	H1-hESC_3
ATF1	K562	NEUROD1	K562, MCF-7
ATF2	GM12878, H1-hESC_3, HepG2, K562	NFATC1	GM12878
ATF3	GM12878, H1-hESC_3, HCT116, HepG2, K562	NFATC3	GM12878
ATF4	K562	NFATC3	K562
ATF7	GM12878, HepG2, K562, MCF-7	Nfe2	GM12878, K562
BACH1	GM12878, H1-hESC_3, K562	NFE2L2	A549, HeLa-S3, HepG2
BATF	GM12878	NFIB	MCF-7
BCL11A	GM12878	NFIC	GM12878, HepG2, K562, SK-N.
BCL11A	H1-hESC_3	NFXL1	GM12878, K562, MCF-7
BCL3	A549, GM12878	Nfya	GM12878, HeLa-S3, K562
BCLAF1	GM12878, HepG2, K562	Nfyb	GM12878, HeLa-S3, K562
Bdp1	HeLa-S3, K562	NR0B1	K562
BHLHE40	A549, GM12878, HepG2, K562	NR2C1	GM12878, K562
Brf1	HeLa-S3, K562	NR2C2	GM12878, HeLa-S3, HepG2, K562
Brf2	HeLa-S3, K562	NR2F1	GM12878, K562
CBFA2T2	K562	NR2F2	HepG2, K562
CBFA2T3	K562	NR2F6	HepG2, K562
CBFB	GM12878	NR3C1	A549, K562
CEBPB	A549, GM12878, H1-hESC_3, HCT116, HeLa-S3, HepG2, K562, MCF-7	Nrf1	GM12878, H1-hESC_3, HeLa-S3, HepG2, K562, MCF-7, SK-N.
CLOCK	MCF-7	Pax5	GM12878
CREB1	GM12878, H1-hESC_3, HepG2, K562, MCF-7	PAX8	GM12878, MCF-7
CREB3L1	K562	Pbx3	A549, GM12878, SK-N.
CREBBP	K562	PHB2	HepG2, K562
CREM	GM12878, HepG2, K562	PKNOX1	GM12878, K562, MCF-7
CTBP1	K562, MCF-7	POU2F2	GM12878
CTCF	A549, A673, B, CD14+ monocyte_1, DND-41, GM12878, H1-hESC_3, osteoblast, MM.1S, mammary epithelial_1 NHLF, NPC_2, PC-3, PC-9, SK-N., astrocyte, HCT116, HSMM, HUVEC, myotube, HeLa-S3, HepG2, K562, MCF-7, bipolar neuron, cardiac muscle, dermal fibroblast, hepatocyte, keratinocyte	POU5F1	H1-hESC_3, K562

CTCFL	K562	PRDM1	HeLa-S3
DACH1	K562	Rad21	A549, GM12878, H1-hESC_3, HCT116, HeLa-S3, HepG2, K562, MCF-7, SK-N.
DEAF1	K562	RB1	GM12878, K562
DNase1	A673, B, CD14+ monocyte_1, H1-hESC_3, HCT116, HSMM, HUVEC, hepatocyte, keratinocyte, mammary epithelial_1, HeLa-S3, HepG2, K562, MCF-7, MM.1S, NHLF, NPC_2, myotube, osteoblast, dermal fibroblast, PC-3, PC-9, SK-N., astrocyte, bipolar neuron, cardiac muscle	RELB	GM12878
E2F1	HeLa-S3, K562	REST	A549, GM12878, H1-hESC_3, HCT116, HeLa-S3, HepG2, K562, MCF-7, SK-N.
E2F4	GM12878, HeLa-S3, K562	RFX5	A549, GM12878, H1-hESC_3, HeLa-S3, HepG2, K562, MCF-7, SK-N.
E2F6	A549, H1-hESC_3, HeLa-S3, K562	RNF2	A549, H1-hESC_3, HepG2, K562
E2F7	K562	RUNX1	K562
E2F8	GM12878, K562, MCF-7	RUNX3	GM12878
E4F1	GM12878, K562, MCF-7	RXRA	GM12878, H1-hESC_3, HepG2, SK-N.
EBF1	GM12878	SETDB1	K562
Egr1	GM12878, H1-hESC_3, HCT116, K562, MCF-7	SIN3A	A549, GM12878, H1-hESC_3, HCT116, HepG2, K562, MCF-7, SK-N.
ELF1	GM12878, HCT116, HepG2, K562, MCF-7, SK-N.	Sirt6	H1-hESC_3, K562
ELF4	K562	SIX4	MCF-7
ELK1	A549, GM12878, HeLa-S3, K562, MCF-7	SIX5	GM12878, H1-hESC_3, K562
ELK4	HeLa-S3	SMAD1	GM12878, K562
EP300	A549, GM12878, H1-hESC_3, HeLa-S3, HepG2, K562, MCF-7, SK-N.	SMAD2	K562
ESRRA	A549, GM12878, K562, MCF-7	SMAD5	GM12878, K562
ETS1	GM12878, HepG2, K562	SMARCA4	HeLa-S3, K562
ETV4	HepG2	SMARCB1	HeLa-S3, K562
ETV6	GM12878, K562	SOX13	HepG2
EZH2	A673, B, DND-41, GM12878, H1-hESC_3, HCT116, HSMM, HUVEC, hepatocyte, keratinocyte, HeLa-S3, HepG2, K562, MM.1S, NPC_2, PC-3, PC-9, astrocyte, mammary epithelial_1	SOX6	HepG2, K562
FOS	GM12878, HUVEC, HeLa-S3, K562, MCF-7	SP1	GM12878, H1-hESC_3, HCT116, HepG2, K562, MCF-7
FOSL1	H1-hESC_3, HCT116, K562	SP2	H1-hESC_3, HepG2, K562
FOSL2	A549, HepG2, MCF-7, SK-N.	SP4	H1-hESC_3
FOXA1	HepG2, K562, MCF-7	SPI1	GM12878, K562

FOXA2	HepG2	SREBF1	A549, GM12878, K562, MCF-7
FOXK2	GM12878, HepG2, K562, MCF-7	SREBF2	A549, GM12878, HeLa-S3
FOXM1	GM12878, K562, MCF-7, SK-N.	Srf	GM12878, H1-hESC_3, HCT116, HepG2, K562, MCF-7
FOXP1	HepG2	STAT1	GM12878
GABPA	GM12878, H1-hESC_3, HeLa-S3, HepG2, K562, MCF-7, SK-N.	STAT3	GM12878, HeLa-S3
GABPB1	K562	STAT5A	GM12878, K562
Gata1	K562	TAF1	GM12878, H1-hESC_3, HeLa-S3, HepG2, K562, MCF-7, SK-N.
Gata2	HUVEC, K562	TAF7	H1-hESC_3, K562
GATA3	A549, MCF-7, SK-N.	TAL1	K562
GATA4	HepG2	TARDBP	GM12878, HepG2, K562, MCF-7
GMEB1	K562	TBP	GM12878, H1-hESC_3, HeLa-S3, HepG2, K562
GTF2B	K562	TBX21	GM12878
HCFC1	GM12878, HeLa-S3, HepG2, K562, MCF-7	TBX3	HepG2
HDAC1	K562, A549, GM12878, H1-hESC_3, HepG2, K562, MCF-7	Tcf12	GM12878, H1-hESC_3, HepG2, K562, MCF-7, SK-N.
HDAC3	K562	TCF3	GM12878
HDAC6	GM12878, H1-hESC_3, K562	TCF7	GM12878, HepG2, K562
HES1	K562	TCF7L2	HCT116, HeLa-S3, HepG2, K562, MCF-7
HES1	MCF-7	TEAD4	A549, H1-hESC_3, HCT116, HepG2, K562, MCF-7, SK-N.
HMGN3	K562	THAP1	K562
HNF1A	HepG2	THRA	K562
HNF4A	HepG2	TRIM22	GM12878, HepG2, MCF-7
HNF4G	HepG2	TRIM28	K562
HSF1	GM12878, MCF-7	USF1	GM12878, H1-hESC_3, HCT116, HepG2, K562, SK-N.
IKZF1	GM12878, HepG2, K562	USF2	A549, GM12878, H1-hESC_3, HeLa-S3, HepG2, K562, SK-N.
IKZF2	GM12878	XRCC4	K562
IRF2	K562	YBX1	GM12878, HepG2, K562, MCF-7
IRF3	GM12878, HeLa-S3, HepG2, SK-N.	YBX3	K562
IRF4	GM12878	Yy1	GM12878, H1-hESC_3, HCT116, HepG2, K562, SK-N.
IRF5	GM12878	ZBED1	GM12878, K562
JUN	A549, H1-hESC_3, HUVEC, HeLa-S3, HepG2, K562, MCF-7	ZBTB33	GM12878, HCT116, HepG2, K562, MCF-7, SK-N.
Junb	A549, GM12878, K562	ZBTB7A	HepG2, K562

Jund	GM12878, H1-hESC_3, HCT116, HeLa-S3, HepG2, K562, MCF-7, SK-N.	ZBTB7B	MCF-7
KDM1A	A549, GM12878, H1-hESC_3, HepG2, K562	ZC3H11A	A549, K562
KDM5A	A549, H1-hESC_3	ZEB1	GM12878, HepG2
KDM5B	K562	ZHX1	HeLa-S3, K562
KLF16	K562	ZHX2	HepG2, MCF-7
KLF5	GM12878	ZKSCAN1	HeLa-S3, HepG2, K562, MCF-7
LEF1	K562	ZNF207	GM12878, HepG2, MCF-7
MAFF	HeLa-S3, HepG2, K562	ZNF217	GM12878, MCF-7
MAFK	A549, GM12878, H1-hESC_3, HeLa-S3, HepG2, K562, MCF-7	ZNF24	GM12878, HepG2, K562, MCF-7
Max	A549, GM12878, H1-hESC_3, HCT116, HUVEC, HeLa-S3, HepG2, K562, MCF-7, SK-N.	Znf263	K562
MEF2A	GM12878, K562, SK-N.	ZNF274	GM12878, H1-hESC_3, HCT116, HeLa-S3, HepG2, K562
MEF2B	GM12878	ZNF444	MCF-7
MEF2C	GM12878	ZNF639	K562
MEIS2	K562	ZSCAN29	GM12878, K562
MGA	K562	ZZZ3	GM12878, HeLa-S3, K562
MITF	K562	MGA	K562
MNT	HepG2, K562, MCF-7	MITF	K562
MYBL2	HepG2, K562		

Table S2. SIAE promoter (ENSR00000046429) transcription factor binding sites (TFBS). SIAE promoter (ENSR00000046429) TFBS deposited in ENCODE database according to ENSEMBL and filtering by Open chromatin & TFBS as Evidence Type [ref.

Transcription Factor	Cell type	Transcription Factor	Cell type
AFF1	K562	MITF	K562
ARID3A	GM12878, HepG2, K562, MCF-7	MNT	HepG2, K562, MCF-7
ARNT	GM12878, HepG2, K562	MYBL2	HepG2, K562
ATF1	K562	MYC	A549, GM12878, H1-hESC_3, HUVEC, HeLa-S3, HepG2, K562, MCF-7
ATF2	GM12878, H1-hESC_3, HepG2, K562	MYNN	K562
ATF3	GM12878, H1-hESC_3, HCT116, HepG2, K562	Nanog	H1-hESC_3
ATF4	K562	NEUROD1	K562, MCF-7
ATF7	GM12878, HepG2, K562, MCF-7	NFATC1	GM12878
BACH1	GM12878, H1-hESC_3, K562	NFATC3	GM12878, K562
BATF	GM12878	Nfe2	GM12878, K562
BCL11A	GM12878, H1-hESC_3	NFE2L2	A549, HeLa-S3, HepG2
BCL3	A549, GM12878	NFIB	MCF-7
BCLAF1	GM12878, HepG2, K562	NFIC	GM12878, HepG2, K562, SK-N.
Bdp1	HeLa-S3, K562	NFXL1	GM12878, K562, MCF-7

BHLHE40	A549, GM12878, HepG2, K562	Nfya	GM12878, HeLa-S3, K562
Brf1	HeLa-S3, K562	Nfyb	GM12878, HeLa-S3, K562
Brf2	HeLa-S3, K562	NR0B1	K562
CBFA2T2	K562	NR2C1	GM12878, K562
CBFA2T3	K562	NR2C2	GM12878, HeLa-S3, HepG2, K562
CBFB	GM12878	NR2F1	GM12878, K562
CEBPB	A549, GM12878, H1-hESC_3, HCT116, HeLa-S3, HepG2, K562, MCF-7	NR2F2	HepG2, K562, MCF-7
CLOCK	MCF-7	NR2F6	HepG2, K562
CREB1	GM12878, H1-hESC_3, HepG2, K562, MCF-7	NR3C1	A549, K562
CREB3L1	K562	Nrf1	GM12878, H1-hESC_3, HeLa-S3, HepG2, K562, MCF-7, SK-N.
CREBBP	K562	Pax5	GM12878
CREM	GM12878, HepG2, K562	PAX8	GM12878, MCF-7
CTBP1	K562, MCF-7	Pbx3	A549, GM12878, SK-N.
CTCF	A549, A673, B, CD14+ monocyte_1, DND-41, GM12878, H1-hESC_3, HCT116, HSMM, HUVEC, HeLa-S3, HepG2, K562, MCF-7, MM.1S, NHLF, NPC_2, PC-3, PC-9, SK-N., astrocyte, bipolar neuron, cardiac muscle, dermal fibroblast, hepatocyte, keratinocyte, mammary epithelial_1, myotube, osteoblast	PHB2	HepG2, K562
CTCFL	K562	PKNOX1	GM12878, K562, MCF-7
DACH1	K562	POU2F2	GM12878
DEAF1	K562	POU5F1	H1-hESC_3, K562
DNase1	A673, B, CD14+ monocyte_1, H1-hESC_3, HCT116, HSMM, HUVEC, HeLa-S3, HepG2, K562, MCF-7, MM.1S, NHLF, NPC_2, PC-3, PC-9, SK-N., astrocyte, bipolar neuron, cardiac muscle, dermal fibroblast, hepatocyte, keratinocyte, mammary epithelial_1, myotube, osteoblast	PRDM1	HeLa-S3
E2F1	HeLa-S3, K562	Rad21	A549, GM12878, H1-hESC_3, HCT116, HeLa-S3, HepG2, K562, MCF-7, SK-N.
E2F4	GM12878, HeLa-S3, K562	RB1	GM12878, K562
E2F6	A549, H1-hESC_3, HeLa-S3, K562	RELB	GM12878
E2F7	K562	REST	A549, GM12878, H1-hESC_3, HCT116, HeLa-S3, HepG2, K562, MCF-7, SK-N.
E2F8	GM12878, K562, MCF-7	RFX5	A549, GM12878, H1-hESC_3, HeLa-S3, HepG2, K562, MCF-7, SK-N.

E4F1	GM12878, K562, MCF-7, GM12878	RNF2	A549, H1-hESC_3, HepG2, K562
Egr1	GM12878, H1-hESC_3, HCT116, K562, MCF-7	RUNX1	K562
ELF1	GM12878, HCT116, HepG2, K562, MCF-7, SK-N.	RUNX3	GM12878
ELF4	K562	RXRA	GM12878, H1-hESC_3, HepG2, SK-N.
ELK1	A549, GM12878, HeLa-S3, K562, MCF-7	SETDB1	K562
ELK4	HeLa-S3	SIN3A	A549, GM12878, H1-hESC_3, HCT116, HepG2, K562, MCF-7, SK-N.
EP300	A549, GM12878, H1-hESC_3, HeLa-S3, HepG2, K562, MCF-7, SK-N.	Sirt6	H1-hESC_3, K562,
ESRRA	A549, GM12878, K562, MCF-7	SIX4	MCF-7
ETS1	GM12878, HepG2, K562	SIX5	GM12878, H1-hESC_3, K562
ETV4	HepG2	SMAD1	GM12878, K562
ETV6	GM12878, K562	SMAD2	K562
EZH2	A673, B, DND-41, GM12878, H1-hESC_3, HCT116, HSMM, HUVEC, HeLa-S3, HepG2, K562, MM.1S, NPC_2, PC-3, PC-9, astrocyte, hepatocyte, keratinocyte, mammary epithelial_1,	SMAD5	GM12878, K562
FOS	GM12878, HUVEC, HeLa-S3, K562, MCF-7	SMARCA4	HeLa-S3, K562
FOSL1	H1-hESC_3, HCT116, K562	SMARCB1	HeLa-S3, K562
FOSL2	A549, HepG2, MCF-7, SK-N.	SOX13	HepG2
FOXA1	HepG2, K562, MCF-7	SOX6	HepG2, K562
FOXA2	HepG2	SP1	GM12878, H1-hESC_3, HCT116, HepG2, K562, MCF-7
FOXK2	GM12878, HepG2, K562, MCF-7	SP2	H1-hESC_3, HepG2, K562
FOXM1	GM12878, K562, MCF-7, SK-N.	SP4	H1-hESC_3
FOXP1	HepG2	SPI1	GM12878, K562
GABPA	GM12878, H1-hESC_3, HeLa-S3, HepG2, K562, MCF-7, SK-N.	SREBF1	A549, GM12878, K562, MCF-7
GABPB1	K562	SREBF2	A549, GM12878, HeLa-S3
Gata1	K562	Srf	GM12878, H1-hESC_3, HCT116, HepG2, K562, MCF-7
Gata2	HUVEC, K562	STAT1	GM12878
GATA3	A549, MCF-7, SK-N.	STAT3	GM12878, HeLa-S3
GATA4	HepG2	STAT5A	GM12878, K562
GMEB1	K562	TAF1	GM12878, H1-hESC_3, HeLa-S3, HepG2, K562, MCF-7, SK-N.
GTF2B	K562	TAF7	H1-hESC_3, K562
HCFC1	GM12878, HeLa-S3, HepG2, K562, MCF-7	TAL1	K562

HDAC1	K562	TARDBP	GM12878, HepG2, K562, MCF-7
HDAC2	A549, GM12878, H1-hESC_3, HepG2, K562, MCF-7	TBP	GM12878, H1-hESC_3, HeLa-S3, HepG2
HDAC3	K562	TBX21	GM12878
HDAC6	GM12878, H1-hESC_3, K562	TBX3	HepG2
HES1	K562, MCF-7	Tcf12	GM12878, H1-hESC_3, HepG2, K562, MCF-7, SK-N.
HMGN3	K562	TCF3	GM12878
HNF1A	HepG2	TCF7	GM12878, HepG2, K562
HNF4A	HepG2	TCF7L2	HCT116, HeLa-S3, HepG2, K562, MCF-7
HNF4G	HepG2	TEAD4	A549, H1-hESC_3, HCT116, HepG2, K562, MCF-7, SK-N.
HSF1	GM12878, MCF-7	THAP1	K562
IKZF1	GM12878, HepG2, K562	THRA	K562
IKZF2	GM12878	TRIM22	GM12878, HepG2, MCF-7
IRF2	K562	TRIM28	K562
IRF3	GM12878, HeLa-S3, HepG2, SK-N.	USF1	GM12878, H1-hESC_3, HCT116, HepG2, K562, SK-N.
IRF4	GM12878	USF2	A549, GM12878, H1-hESC_3, HeLa-S3, HepG2, K562, SK-N.
IRF5	GM12878	XRCC4	K562
JUN	A549, H1-hESC_3, HUVEC, HeLa-S3, HepG2, K562, MCF-7	YBX1	GM12878, HepG2, K562, MCF-7
Junb	A549, GM12878, K562	YBX3	K562
Jund	GM12878, H1-hESC_3, HCT116, HeLa-S3, HepG2, K562, MCF-7, SK-N.	Yy1	GM12878, H1-hESC_3, HCT116, HepG2, K562, SK-N.
KDM1A	A549, GM12878, H1-hESC_3, HepG2, K562	ZBED1	GM12878
KDM5A	A549, H1-hESC_3	ZBED1	K562
KDM5B	K562	ZBTB33	GM12878, HCT116, HepG2, K562, MCF-7, SK-N.
KLF16	K562	ZBTB7A	HepG2, K562
KLF5	GM12878	ZBTB7B	MCF-7
LEF1	K562	ZC3H11A	A549, K562
MAFF	HeLa-S3, HepG2, K562	ZEB1	GM12878, HepG2
MAFK	A549, GM12878, H1-hESC_3, HeLa-S3, HepG2, K562	ZHX1	HeLa-S3, K562
Max	A549, GM12878, H1-hESC_3, HCT116, HUVEC, HeLa-S3, HepG2, K562, MCF-7, SK-N.	ZHX2	HepG2, MCF-7
MEF2A	GM12878, K562, SK-N.	ZKSCAN1	HeLa-S3, HepG2, K562, MCF-7
MEF2B	GM12878	ZNF207	GM12878, HepG2, MCF-7
MEF2C	GM12878	ZNF217	GM12878, MCF-7
MEIS2	K562	ZNF24	GM12878, HepG2, K562, MCF-7
MGA	K562	Znf263	K562
